# Mouse models for bacterial enteropathogen infections: insights into the role of colonization resistance

**DOI:** 10.1080/19490976.2023.2172667

**Published:** 2023-02-16

**Authors:** Mathias K.-M. Herzog, Monica Cazzaniga, Audrey Peters, Nizar Shayya, Luca Beldi, Siegfried Hapfelmeier, Markus M. Heimesaat, Stefan Bereswill, Gad Frankel, Cormac G.M. Gahan, Wolf-Dietrich Hardt

**Affiliations:** a Department of Biology, Institute of Microbiology, ETH Zurich, Zurich, Switzerland; b APC Microbiome Ireland, University College Cork, Cork, Ireland; c School of Microbiology, University College Cork, Cork, Ireland; d Department of Life Sciences, MRC Centre for Molecular Bacteriology and Infection, Imperial College London, London, UK; e Institute of Microbiology, Infectious Diseases and Immunology, Charité - University Medicine Berlin, Berlin, Germany; f Institute for Infectious Diseases, University of Bern, Bern, Switzerland; g School of Pharmacy, University College Cork, Cork, Ireland

## Abstract

Globally, enteropathogenic bacteria are a major cause of morbidity and mortality.^1-3^ *Campylobacter, Salmonella*, Shiga-toxin-producing *Escherichia coli*, and *Listeria* are among the top five most commonly reported zoonotic pathogens in the European Union.^4^ However, not all individuals naturally exposed to enteropathogens go on to develop disease. This protection is attributable to colonization resistance (CR) conferred by the gut microbiota, as well as an array of physical, chemical, and immunological barriers that limit infection. Despite their importance for human health, a detailed understanding of gastrointestinal barriers to infection is lacking, and further research is required to investigate the mechanisms that underpin inter-individual differences in resistance to gastrointestinal infection. Here, we discuss the current mouse models available to study infections by non-typhoidal *Salmonella* strains, *Citrobacter rodentium* (as a model for enteropathogenic and enterohemorrhagic *E. coli), Listeria monocytogenes*, and *Campylobacter jejuni. Clostridioides difficile* is included as another important cause of enteric disease in which resistance is dependent upon CR. We outline which parameters of human infection are recapitulated in these mouse models, including the impact of CR, disease pathology, disease progression, and mucosal immune response. This will showcase common virulence strategies, highlight mechanistic differences, and help researchers from microbiology, infectiology, microbiome research, and mucosal immunology to select the optimal mouse model.

## Introduction

The intestine plays a key role in the digestion of food and absorption of nutrients and is the location of a significant proportion of the immune system in higher animals.^1^, ^2^, ^3^, ^4^, ^5,6^ It is directly exposed to the external environment and is therefore at significant risk of infection. The gastrointestinal tract houses a very dense microbial community, the gut microbiota, which aids digestion, immune conditioning, and host defense.^6–8^ At higher taxonomic levels, the microbiota community structure is similar between different mammalian species^9,10^ as they are generally composed of 4–6 major phyla (most prevalently Bacteroidetes, Firmicutes, Proteobacteria, Verrucomicrobiota, and Actinobacteria), suggesting that animal models are generally suitable for investigating mechanisms that control microbial infection in mammals.^10^ Moreover, the adult microbiota community structure is relatively stable over time,^11^ indicating that most microbial species continuously ingested via food or water are prevented from being established in the gut.

The gut is the site of infection for a diverse range of enteropathogenic bacteria, including Gram-negative Enterobacteriaceae such as *Salmonella enterica*, enteropathogenic *Escherichia coli* (EPEC), enterohemorrhagic *E. coli* (EHEC), or *Citrobacter rodentium*, the Gram-negative pathogen *Campylobacter jejuni* as well as Gram-positive pathogens such as *Listeria monocytogenes* or *Clostridioides difficile*.^12–15^ In this review, we discuss the similarities and differences between these pathogens and how their infection biology can be studied in murine infection models. We review their control by chemical and physical barriers, the role of colonization resistance (CR), as well as the immunological defense mechanisms, which are mounted by the intestinal mucosa.

### Barriers against enteric infections

Upon ingestion, the acidity of the stomach is a formidable barrier that eliminates the majority of incoming bacteria. Within the intestine, anaerobiosis, antimicrobial peptides, and nutrient scarcity also influence luminal growth of the pathogen. The physical protection provided by the mucus layer covering the gut epithelium^16,17^ and fast gut transit times further reduce the local proliferation of the pathogen.^18–21^

**Figure 1.**
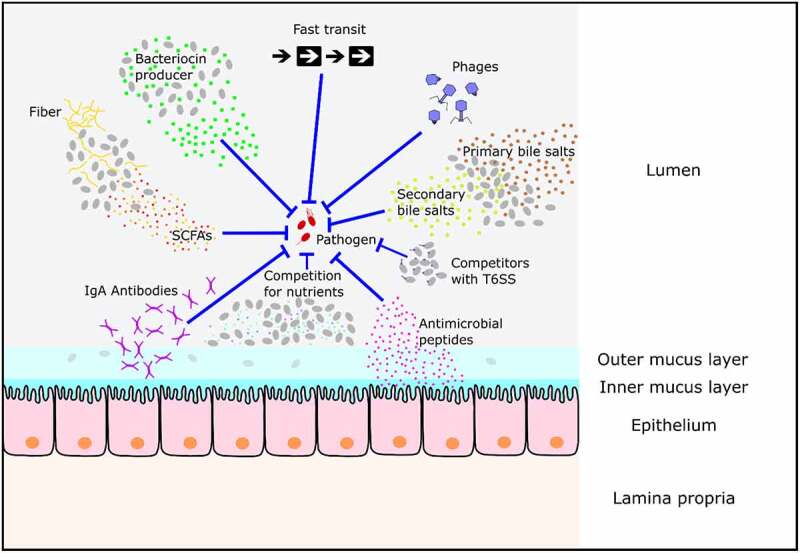
Known mechanisms inhibiting enteropathogen growth in the gut. The resident microbiota in the gut of mammals protects their host from pathogens via mechanisms including the production of short-chain fatty acids (SCFAs), the secretion of bacteriocins, the conversion of primary into secondary bile salts, and the competition for nutrients from the host diet. In addition, phages restrict the colonization of invading bacteria. Finally, a fast transit time makes it challenging for a pathogen to colonize the gut and intestinal epithelial cells (IECs) secrete antimicrobial peptides and IgA antibodies. It is not known if all of the mechanisms indicated are relevant to every enteropathogen.

The protection conferred by the resident microbiota, referred to as “colonization resistance” (CR), is the result of multiple factors including the production of antimicrobial substances, nutrient competition, and bacteriophage activity (see Figure 1).^22–24^ It is estimated that about 25% of bacteria in the human colon carry genes for the production of type VI secretion systems (T6SS) which they may use for the direct killing of invading pathogens.^25^ Antimicrobial products such as bacteriocins are produced by certain bacteria (Gram-positive and Gram-negative species alike) to limit the growth of competitors.^26^ Bacteriocins act by inhibiting cell wall biosynthesis, transcription, translation, DNA replication, and outer membrane biogenesis, as well as by disrupting cell membranes.^22,26^ In addition, nutrient competition is regarded as one of the most important factors when it comes to the establishment of a new bacterium in the gut.^27,28^ Microorganisms in the gut compete for macro- and micronutrients provided by the host diet, intestinal epithelial cells, and the resident microbiota (cross-feeding).^28–30^ Finally, bacteriophages are highly specific bacterial viruses which often target a limited number of bacterial strains of given species.^31^ However, the human phageome is highly diverse and stably covers a wide range of bacterial species and has been shown to contribute to the exclusion of incoming microorganisms in the gut.^32^ Together, these mechanisms determine if an incoming pathogen will prevail, bloom, and eventually cause disease.

In addition to overcoming CR, an invading pathogen must avoid, escape, or endure the adaptive and innate immune responses of the host. The mucosal immune system faces a challenge to distinguish invading pathogens from commensal organisms while maintaining homeostasis, promoting appropriate immune responses, and limiting immune-mediated damage. IECs play a vital role not only as a physical barrier but also as a means of communication between the microbiota and the host.^33,34^ They secrete mucins, antimicrobial peptides, and hormones into the gut lumen and communicate with immune cells on the basolateral side.^34^ Furthermore, neutrophilic granulocytes can transmigrate into the gut lumen and attack bacteria by phagocytosis, release of antimicrobial substances, and the formation of neutrophil extracellular traps (NETs).^35–38^ Intestinal antibodies (mainly of the IgA isotype) specifically restrict the colonization of certain bacterial strains by blocking their access to epithelial receptors, entrapping them in mucus, and facilitating their removal by peristaltic activity,^39,40^ or by selectively clumping rapidly dividing bacteria.^39^

These barriers are so effective that many hosts will remain healthy upon exposure to an enteropathogenic bacterium. However, some individuals will develop enteric disease. It is currently unclear which mechanisms define a successful barrier to infection though significant progress has been made in the investigation of individual responses to several pathogens, most notably *C. difficile*.^22,41–43^ CR can be disrupted by various drugs such as antibiotics, proton pump inhibitors, antidiabetics, and antipsychotics as well as by dietary shifts.^22,23,44,45^ Some of these substances can be utilized in mouse models to break CR and enable researchers to reproducibly study mechanistic factors which influence enteric diseases. Since efficient therapies or vaccines against most enteric bacterial infections are still lacking, research into the disease mechanisms and the mechanisms that prevent acute infections is of great importance.

### Why do we still need animal infection models?

In the intestine, enteropathogenic bacteria engage in complex and incompletely understood interactions with the microbiota, food and its digestion products, the intestinal mucosa, and the host’s immune system. The *in vitro* models available today insufficiently recapitulate this complexity of interactions. Thus, animal models remain necessary for studying host–enteropathogen infections. Advantages and limitations of mouse models in research on the gastrointestinal tract and the microbiome as a proxy for its human counterpart have been extensively reviewed in Nguyen et al. 2015 and Hugenholtz et al. 2018^10,46^ Our review will summarize and compare the specific features of several available mouse gut infection models. We will cover mouse models for enteric disease triggered by *C. jejuni, C. difficile*, EPEC, EHEC, *L. monocytogenes*, and *S. enterica* serovar Typhimurium (*S*. Tm) to foster research on filling in the gaps in our knowledge about enteric infections.

## Mouse models for enteropathogen infection

### Non-typhoidal *Salmonella enterica*

*S. enterica* causes 180 million cases of diarrheal disease globally each year.^47^ The most prevalent non-typhoidal *S. enterica* serovars diagnosed in human diarrhea are Enteritidis and Typhimurium (*S*. Tm).^1^ Humans are commonly infected by contaminated foods, most notably eggs, pork, poultry meat, and dairy products.^1^ Disease symptoms usually begin 7–132 h after the ingestion of contaminated food^48^ and include abdominal pain (gastroenteritis), diarrhea, nausea, sometimes vomiting, and transient fever.^1^ In healthy individuals, the infection is self-limiting, acute diarrhea ends after 3–5 d, and pathogen-shedding in the stool will last for a few more weeks. Even though systematic data are scarce, the infection is typically associated with intestinal inflammation and increases the risk of irritable or inflammatory bowel disease.^49^ Systemic spread associated with fever can occur in the young, the elderly, and immunocompromised people. The incidence of the disease is 10- to 100-fold lower than the rate of exposure,^23^ but the risk of infection increases after an antibiotic treatment.^50,51^ In combination with our knowledge from mouse models (discussed below), this suggests the importance of protection by CR.

Mouse infection models have revealed a key protective role of the gut microbiota. Experimental mice with a complex (but specific pathogen-free, so-called “SPF”) microbiome are in most cases resistant against *S*. Tm infection.^44,52^ The CR of SPF mice can be alleviated by antibiotics such as streptomycin, ampicillin, or ciprofloxacin.^52–55^ SPF mice pretreated with 20 mg streptomycin 24 h before infection with 5 × 10^7^ CFU *S*. Tm develop gut inflammation with very little mouse-to-mouse variability. The CFU/g feces reach 10^7^ as early as 8 h postinfection (p.i.), rise to 10^9^ CFU/g by 24 h p.i., and stay at that level for weeks to months.^23,52,56,57^ Gnotobiotic mice associated with up to 12 different microbiota strains (OligoMM^12^ mice) show partial CR.^58^ Infection kinetics are different compared to the streptomycin pretreatment model and gut inflammation appears only after 2–3 d of infection.^23,59^ In the OligoMM^12^ model, colonization resistance against *S*. Tm can be fully restored by adding three additional anaerobic strains to the microbiome.^60^ Moreover, this model offers a unique opportunity to analyze the entire microbiome throughout the infection as all strains are culturable, genetically accessible, and genome sequenced.^60,61^ Mouse-to-mouse variation in the gnotobiotic mouse models is moderate compared to streptomycin-pretreated or germ-free mice.^60,62^ Germ-free mice are fully susceptible to *S*. Tm infection and develop symptoms in the initial 10 h after infection, with a rapid increase in CFU/g feces.^62^ Admittedly, germ-free mice differ from all the above-mentioned mouse models not only in the absence of microbiota but also in the accompanying immaturity of the immune system, which might in part explain the rapid disease progression.^63–65^ However, the mechanisms of *S*. Tm pathogenicity appear to be similar in germ-free and streptomycin-pretreated mice.^62^ Recently, it has been found that dietary composition has pronounced effects on CR against *S*. Tm.^44^ As long as mice harboring a complex SPF microbiota are kept on a standard plant-based mouse chow, they typically exhibit a high degree of CR.^44^ This CR is strongly impaired, if the mice are shifted for as little as 1 d to a western-style diet (high-fat, low-fiber) or a low-fat plus low-fiber diet.^44^ This dietary shift alone (without antibiotic treatment) is sufficient to trigger the disease in most mice after inoculation with the same inoculum size (5 x 10^7^ CFU).^44^ Evidence suggests that altered food composition creates a transient niche for colonization. In the case of a high-fat diet, the compromised CR was traced back to enhanced bile salt secretion.^44^ This physiological response aids fat digestion, but high primary bile salt concentrations inhibit microbiota growth, while *S*. Tm can tolerate up to 10-fold higher bile salt concentrations than other members of the microbiota.^44^ Altered enterocyte physiology, as indicated by changes in mucosal gene expression and Enterobacteriaceae colonization experiments, may further contribute to reduced CR.^66,67^ In the high-fat diet model for *S*. Tm gut infection, the microbiota is only mildly suppressed (compared to antibiotic-pretreated mice) and overt enteropathy takes at least 48 h to develop.^23,44^ The mouse-to-mouse variation with this model is rather high, but in most mice, *Salmonella* stool densities reach 10^9^ CFU/g after 72 h of infection.^23,44^ Finally, in other studies, newborn mice were found to lack CR and permit-efficient gut luminal growth of *S*. Tm and pathogen spread to systemic sites.^68^

Despite the availability of the above-mentioned mouse models with various levels of CR and decades of research in the microbiota field,^69^ further work is necessary to identify the precise constituents of a functional microbiome needed to protect against *Salmonella* spp. infections. Nevertheless, these mouse models have facilitated important insights into the mechanisms of gut colonization by *S*. Tm and have consolidated the fundamental concept of CR against *Salmonella* spp.

**Figure 2.**
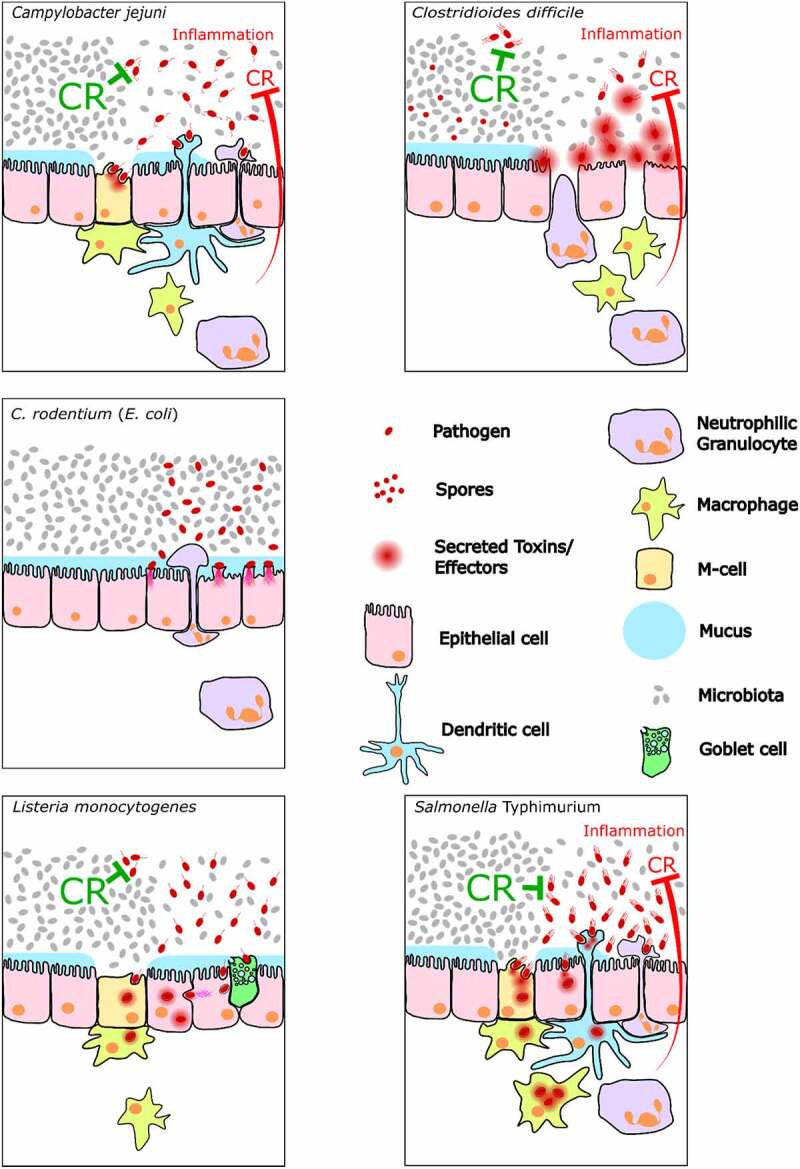
Comparison of the infection process at the intestinal epithelium between the five enteropathogens. The red inhibition arrow indicates the reduction in CR against the pathogen if the pathogen can trigger gut inflammation.

*S*. Tm infections in microbiota-colonized mice have shown that the pathogen initially grows by anaerobic hydrogen/fumarate respiration, thus utilizing leftover food molecules and intermediates from anaerobic microbiota metabolism.^28,59,70^ The pathogen employs flagella to penetrate through gaps in the mucus layer^16^ and *cheY, motA, fliC*, and *fliB* mutants of *S*. Tm show delayed disease kinetics in streptomycin-pretreated mice.^71,72^ Upon arrival at the epithelial surface, *S*. Tm uses adhesins and the TTSS-1, a syringe-like protein injection system to trigger invasion into the gut epithelium.^52,73–75^ Furthermore, it employs TTSS-2 to traverse the epithelium and proliferate in the lamina propria and organized tissues of the gut-associated immune system (Figure 2).^76^ The tissue-lodged bacteria and their products (such as LPS^77^) trigger further innate immune responses. Gut inflammation has both a detrimental and a beneficial effect on the colonization of *S*. Tm in the gut.^35^ Severe forms of gut inflammation, as observed in the streptomycin mouse model, can eradicate as much as 99.999% of the gut luminal *S*. Tm population. However, inflammation also provides the surviving pathogens with a relative growth advantage against the resident microbiota.^35,56^ Factors that contribute to this selective advantage include the availability of host-derived oxygen and other respiratory electron acceptors, such as tetrathionate and nitrate.^78–81^ 129SvEv mice (expressing a functional natural resistance-associated macrophage protein (NRAMP) which enables them to remove divalent metal ions from the pathogen-containing vacuole) can control systemic infection and can be used to study long-term infections with wild-type *Salmonella* spp.^57,82,83^ As C57BL/6 and Balb/c mice carry an NRAMP mutation, they cannot efficiently control systemic pathogen spread.^57,82^ To prevent life-threatening stages of systemic disease, gut infection assays with wild-type *S*. Tm in these mice are typically limited to 4 d. Despite this limitation, the extensive set of knockout lines, analytic tools, and the large body of immunological literature on C57BL/6 mice has driven the discovery of mechanisms using this model, in particular in streptomycin-pretreated C57BL/6 mice. Here, gut-luminal growth is associated with invasion into the gut epithelium, as well as proliferation in the gut-associated lymphatic tissue and the lamina propria. This tissue invasion triggers innate immune responses, including the epithelial NAIP/NLRC4 inflammasome,^75,84–88^ Interleukin (IL)-18 release by the gut epithelium,^89^ Tumor Necrosis Factor (TNF)-production by lamina propria cells,^77,85^ Interferon gamma (IFNγ)-elicited triggering of mucus secretion by goblet cells^90^ and expulsion of infected enterocytes into the gut lumen.^84^ Despite differences in the way that CR is alleviated, *S*. Tm infections in adult mice appear to elicit quite similar enteropathy. This is in striking contrast to infections in newborn mice, where the pathogen invades the gut tissue, but fails to trigger overt enteropathy or the expulsion of infected enterocytes.^68^ The molecular and cellular differences between the disease in newborn and adult mice are still not fully understood.

In adult mice, expulsion of infected enterocytes and infiltration by neutrophilic granulocytes pose a strong defense mechanism against *S*. Tm. The neutrophilic granulocytes transmigrate into the gut lumen and substantially diminish the pathogen population in the gut during inflammation.^35,91,92^ Attenuated *S*. Tm mutants defective in systemic spread in C57BL/6 mice (and 129SvEv mice) permit the analysis of the adaptive immune response by d 10–30 p.i.^93,94^ This includes the development of protective antibody responses against flagellins, outer membrane porins, and the lipopolysaccharide (LPS) O-antigen.^95–97^ O-antigen-specific secretory IgA, “enchains” gut luminal *Salmonella* cells (leading to the formation of monoclonal pathogen clumps), which prevents further tissue invasion and accelerates pathogen elimination from the gut.^39,94^ While a complex microbiota that lack *E. coli* strains can still confer substantial CR (e.g. by propionate-mediated disruption of the pathogen’s pH homeostasis^98^), *E. coli* strains have been identified as important in outcompeting *S*. Tm from the diseased gut.^30,44,99,100^ Competition for micronutrients, such as iron ions, terminal electron acceptors like oxygen or nitrate and bacteriocin production, is thought to contribute to this *E. coli*-mediated CR against *Salmonella* infection.^30,78,101,102^

### Campylobacter jejuni

The Gram-negative, spirally curved *C. jejuni* belongs to the four key global causes of food-borne diarrheal infections.^103^ Among other bacterial species within the genus Campylobacter, *C. jejuni* (and less frequently *Campylobacter coli, Campylobacter lari*, and *Campylobacter upsaliensis*) are the most common causative agents of campylobacteriosis. The manifestations of intestinal enteritis range from mild to severe symptoms, which include, but are not limited to, 1–3 d of fever, vomiting, and malaise followed by 3–7 d of abdominal cramps and bloody diarrhea.^104,105^ The initial enteritis is induced by a low dose of *C. jejuni* bacteria that are mainly transmitted by the consumption of raw or undercooked poultry meat, unpasteurized milk, contaminated surface water, as well as cross-contaminated food that is consumed uncooked, such as fruits and vegetables.^106^ With a lower incidence, other non-gastrointestinal sequelae are associated with campylobacteriosis. Systemic manifestations include infectious complications such as bacteremia and post-infectious immune disorders such as reactive arthritis, Guillain–Barré syndrome, and myocarditis.^107,108^ In a small but relevant subpopulation of infected individuals, *C. jejuni* infection triggers the onset of chronic intestinal diseases, such as ulcerative colitis, Crohn´s disease, or irritable bowel syndrome.^109,110^ Clinical studies revealed that it is fundamental to the understanding of pathogenesisinduced by *C. jejuni* infection that both the severity of initial enteritis and the risk for the development of post-infectious syndromes are dependent on the molecular structure of the surface endotoxin lipooligosaccharide (LOS), which is highly variable among individual *C. jejuni* strains.^111–113^ Thus, endotoxin-induced inflammation initiated by innate immune responses determines the severity of symptoms and the subsequent complications of campylobacteriosis.^114^

The commensal murine microbiota provides full CR against *C. jejuni*, thus preventing colonization of the gastrointestinal tract of conventional laboratory mice. For many years, this precluded the development of proper murine infection models for campylobacteriosis. Effective colonization of the murine intestinal tract by *C. jejuni* requires modifications to the composition of the gut microbiota. This can be accomplished by aggressive antibiotic treatment that yields so-called “secondary abiotic” (SAB) wild-type mice, which can be colonized by *C. jejuni* but lack overt clinical signs of infection and campylobacteriosis symptoms.^108^ In the gut of C3H mice colonized by a limited defined enteric microbiota consisting of nonpathogenic Clostridial species, *Lactobacillus*, and *Acinetobacter, C. jejuni* was able to colonize at high loads reaching concentrations of 10^8^ to 10^9^ CFU/g of feces after 1 week of infection. Infection doses as little as 200 bacteria were sufficient for intestinal colonization, while the individual immune responses of mice were highly variable.^115^ Infant mice constitute another murine campylobacteriosis model.^116–120^ Immediately after weaning, conventional 3-week-old wild-type mice develop self-limited enterocolitis characterized by bloody diarrhea and colonic epithelial cell apoptosis within 1 week following *C. jejuni* infection and recovered thereafter, thus mimicking the time course of human campylobacteriosis.^119,120^ Gut microbiota analyses revealed that infant mice harbored higher commensal *E. coli* but lower Lactobacilli numbers in their large intestines as compared to adult mice.^120^ The elevated colonic *E. coli* loads might explain the susceptibility of infant mice toward *C. jejuni* colonization given that exogenous application of commensal *E. coli* to conventional adult wild-type mice was shown to abrogate the CR against *C. jejuni*.^120^

Interestingly, SAB wild-type mice reconstituted with human gut microbiota derived from healthy donors left these mice susceptible to *C. jejuni* infection. This was not the case with SAB mice recolonized with murine gut microbiota. *C. jejuni* loads in mice associated with human gut microbiota reached high levels of 10^8^ CFU/g of feces in the 1^st^ d after infection which remained constant for weeks. However, *C. jejuni* colonization was effectively cleared during the first 2–3 d after infection in mice associated with murine gut microbiota.^104^ These studies demonstrate that CR depends on distinct microbial communities that exist in the murine intestinal microbiota. Therefore, SAB mice and other murine models of infection, wherein antibiotics are used to perturb the gut microbiota and abolish CR, have been efficiently used for the study of intestinal colonization by *C. jejuni*. Oral vancomycin treatment, which promotes *S*. Tm colonization in a similar fashion to streptomycin pretreatment,^121^ was shown to deplete bacteroidetes and clostridia and to increase *Lactobacillus* populations.^122^ In vancomycin-pretreated mice, *C. jejuni* was able to establish intestinal colonization at high levels,^123^ and this was also the case upon pretreatment of mice with ciprofloxacin or penicillin at therapeutic dosages.^124^ Similarly, ampicillin-pretreated CBA/J mice were shown to be susceptible to *C. jejuni* colonization.^125^

As a consequence of antibiotic treatment, SAB wild-type mice can be colonized by *C. jejuni* but do not develop infectious enteritis, mainly because the murine innate immune system does not react against the LOS of *C. jejuni*.^126^ This was shown by Mansfield et al., who successfully developed a murine campylobacteriosis model, in which the absence of IL-10 drastically increases the LOS sensitivity of mice and impairs the immune system’s capacity to resolve inflammation.^127^ However, this work was done in mice that overexpressed innate immune receptors.^126^ Finally, the SAB IL-10-deficient mice were confirmed to reproducibly develop typical symptoms of acute campylobacteriosis upon *C. jejuni* infection. These mice were successfully used to study the immunopathological response against defined *C. jejuni* virulence factors during disease initiation and progression.^128–131^ Moreover, further standardization of *C. jejuni* infection in SAB IL-10-deficient mice allowed for preclinical studies testing the efficacy of drugs affecting the innate immune response against campylobacteriosis.^109^

Compared to other enteric bacterial pathogens, *C. jejuni* does not rely on conventional exotoxins that are typically utilized to infect the host. Consequently, campylobacteriosis constitutes an endotoxin-mediated inflammatory disease induced by the contact of *C. jejuni*-LOS and other endotoxins with innate immune cells, such as dendritic cells, macrophages, monocytes, and neutrophilic granulocytes. However, the physical contact between live *C. jejuni* and host immune cells depends on the ability of the pathogen to move into the mucus layer, adherence to epithelial cells, and subsequent invasion to subepithelial tissue sites. This is accomplished by the flagellum present at one or at both ends of the cell,^132^ by adhesins, and by invasins (Figure 2).^133^ In addition to their function in bacterial motility, *C. jejuni* utilizes flagella for protein secretion, biofilm formation, and adhesion.^134,135^ Flagellar motility of *C. jejuni* is associated with a chemotaxis system, which is essential for effective colonization in the avian and mammalian guts. *C. jejuni* displays a chemotactic response to amino acids and organic acids originating from either the host or the residual gut microbiota, in addition to distinct constituents of bile and mucus.^136^

Another important virulence factor for *C. jejuni* is the capsular polysaccharide (CPS), which plays a major role in systemic infection. The structure of the CPS is variable among *C. jejuni* strains and may differ in sugar composition and linkage.^137,138^ CPS also plays an immunomodulatory role by preventing excessive production of cytokines by the host immune system.^139,140^ Moreover, lipid A in LOS helps *C. jejuni* to survive hostile environments, to evade the host immune system, and to adhere to and invade epithelial cells.^141,142^ Interestingly, LOS is not only a major factor in *C. jejuni*-induced intestinal inflammation but is also essential for triggering post-infectious sequelae, such as Guillain–Barré syndrome caused by cross-reactive LOS-specific antibodies as a result of structural mimicry of pathogenic LOS and the surface of neuronal gangliosides.^143^ The use of genetically modified SAB mice as a preclinical model for severe human campylobacteriosis constitutes a major advance for investigating *C. jejuni* virulence factors *in vivo*. As highlighted in the previous paragraph, IL-10-deficient mice provide a valuable tool to investigate differences between LOS variants in the pathogenesis of *C. jejuni*. In addition, the SAB IL-10-deficient mouse model was successfully used to explore the role of the flagella during campylobacteriosis, whereby *C. jejuni* lacking the flagella genes *flaA* and *flaB* were unable to trigger enteric disease despite colonizing the murine colon at high loads.^131^ This model was also applied to investigate the function of other virulence factors, such as the serine protease HtrA and the outer membrane adhesin Cj0268, in *C. jejuni* pathogenesis.^129,144,145^ Moreover, it is valuable for preclinical intervention studies and has already been applied to investigate antibiotic-independent therapeutic approaches, such as using the phenolic compounds carvacrol, curcumin, and resveratrol, as well as vitamin C and D, urolithin A, and activated charcoal, to combat *C. jejuni* or to ameliorate disease manifestations and progression.^146–151^

### *Citrobacter rodentium* as a model for enteropathogenic *Escherichia coli* and enterohaemorrhagic *Escherichia coli*

Human gastrointestinal pathogens EPEC and EHEC remain a major global health problem. While EPEC causes diarrhea in children in low- and middle-income countries, EHEC is mainly found in industrial countries and can lead to hemorrhagic colitis and hemolytic–uremic syndrome (HUS).^152,153^ Several animal models have been used to study EPEC and EHEC *in vivo*.^154^ EPEC has been shown to infect rabbits ^155^ and pigs,^155,156^ and studies of EHEC pathogenesis *in vivo* have involved rabbits,^157^ chickens,^158^ gnotobiotic piglets,^159,160^ and calves.^161^ However, small animal models, and particularly mouse models, exhibit many advantages, including low relative costs of maintenance and the possibility to manipulate host genetics.^162^ Thus, various mouse models were also proposed to study EPEC^163–165^ and EHEC^166–169^ infections. Although there have been some exceptions,^163,169^ studies have mostly shown that EPEC and EHEC do not colonize the mouse intestine in the presence of an intact commensal microbiota.^170^ This is similar to *S*. Typhimurium and *C. jejuni*, as discussed above. In the case of EPEC and EHEC, gnotobiotic,^168^ antibiotic-pretreated,^165–167^ or young mice harboring immature microbiota^164^ are used, as they present a reduced CR against these pathogens. Nevertheless, there is an inherent limitation in these models, as they do not reflect natural and physiological host–pathogen–microbiota interactions.^171^ *C. jejuni* can colonize mice associated with human (but not mouse) microbiotas, as discussed above. Since commensals play a dual role in *C. rodentium* infection, both assisting and repelling the pathogen, it might therefore be of interest to test susceptibility to EPEC/EHEC infection in mice reconstituted with a human microbiota.

*Citrobacter rodentium* is a mouse-adapted pathogen and the etiologic agent of transmissible murine colonic crypt hyperplasia, which causes epithelial cell hyperproliferation and colonic crypt elongation in laboratory mice.^172^ Genomic analysis revealed around 32% of the *C. rodentium* genome is not shared with EPEC and EHEC. Nonetheless, *C. rodentium*, EPEC, and EHEC share a similar infection strategy and virulence genes.^173^ In fact, like EPEC and EHEC infections, colonization of the gastrointestinal tract by *C. rodentium* relies on attaching and effacing (A/E) lesions. These are characterized by intimate attachment of the bacteria to the apical surface of epithelial cells, effacement of the brush border, and formation of actin pedestals beneath the adherent bacteria (Figure 2).^172,174^ The ability of these pathogens to form A/E lesions relies on the locus of enterocyte effacement (LEE) pathogenicity island, which encodes gene regulators, the outer membrane bacterial adhesin intimin, a T3SS and several effector proteins.^175–177^ Additional T3SS effectors are encoded on prophages and insertion sequences. Intimate attachment is mediated by avid interactions between the intimin and the translocated receptor Tir, a pathogen-encoded protein, which is transported into the host cell via the T3SS.^178–181^ Attachment to the IECs triggers a type 3 immune response in the lamina propria.^174,182,183^ During the early stages of infection, *C. rodentium* is recognized by myeloid differentiation primary-response protein 88 (MYD88)-dependent Toll-like receptor (TLR) signaling, mediated by TLR2 and TLR4 at the surface of epithelial cells.^182^ Dendritic cells (DCs) produce IL-23, which induces secretion of IL-22 and IL-17 by type 3 innate lymphoid cells (ILC3s). IL-22 induces IECs to express and secrete antimicrobial peptides, like REG3β and REG3γ, and nutritional immunity proteins, such as calprotectin and lipocalin 2 (LCN2).^174,182^ Consistently, KO mice lacking IL-22, NLRC4, INFγ, TLRs, or Nfil3 (leading to diminished mucosal ILC3) and RAG-1-deficient mice either succumb to *C. rodentium* infection or exhibit severe pathological mucosal damage.^184,185^ As a natural A/E mouse pathogen, *C. rodentium* colonizes mice with an intact microbiota,^170^ and therefore provides an ideal model for studying A/E pathogens *in vivo*.^162,171,174,182,186^

The severity of *C. rodentium* infections is dependent on the genetic background of the host. While mouse strains like C57BL/6, BALB/c, 129S1/SvImJ, or NIH Swiss present a mild, self-limiting infection, susceptible strains like C3H/HeJ or C3H/HeOuJ succumb to infection 6 to 12 d after *C. rodentium* inoculation.^187^ Although genetic factors such as the expression of *Rspo2* can have a direct influence on strain susceptibility to infection,^188,189^ these do not completely explain the observed differences.^187^ The genetic background can also indirectly influence the intestinal bacterial community,^190^ affecting susceptibility or resistance to colonization and infection.^191–193^ In fact, fecal transplantations of resistant NIH Swiss or C57BL/6 mice into antibiotic-pretreated (thus susceptible) C3H/HeJ or C3H/HeOuJ mice, respectively,^191,192^ reverted susceptibility phenotypes, leading to delayed colonization^192^ and survival rates varying from 70%^192^ to 100%.^191^ Knockout of the vitamin D receptor in mice has also been shown to lead to altered microbiota conferring CR against *C. rodentium*, even when the dysbiotic microbiota was transferred into germ-free mice.^194^ Consistent with the major role of the microbiota in host protection, germ-free mice or mice presenting a simplified microbiota like in the neonatal or the OligoMM^12^ model do not seem to be able to clear *C. rodentium* infection and present with high bacterial loads in feces up to 42 d p.i.^195–200^ When co-housing adult mice with a neonatal microbiota with conventional adult mice, CR is restored and the infection is rapidly cleared,^197^ showing that a fully developed microbiome is essential for pathogen clearance by outcompeting *C. rodentium*.^195,196^

Diet has also been shown to play a major role in shaping the microbiota and thus, enhancing or impairing *C. rodentium* infection. For example, a reduced-fat diet or the addition of dairy products to normal diet-induced protective effects against *C. rodentium* and ameliorated associated pathology.^201,202^ In contrast, a fiber-free diet leads to the bloom of mucus-layer degrading commensals and thereby increases susceptibility to *C. rodentium* infection.^203^ While treatment of mice with fermented dairy products did not affect *C. rodentium* colonization, organ specificity, or A/E lesion formation, it reduced colonic hyperplasia and it prevented the decrease of Ruminococcus and increased Turicibacteraceae (Turicibacter) abundance,^202^ whose decrease has been associated with susceptibility to dextran sodium sulfate-induced colitis.^204^

Microbial diversity in the gut makes it difficult to disentangle individual contributions, meaning that the mechanisms by which CR is achieved are not yet completely understood. However, several commensals have been reported to play a role in CR against *C. rodentium*. When administered to mice prior to *C. rodentium* inoculation, various *Lactobacillus* strains such as *Lactobacillus rhamnosus, Lactobacillus acidophilus*, or *Lactobacillus helveticus* were able to modulate the immune response during *C. rodentium* infection, thereby reducing immune cell infiltration of the lamina propria and colonic crypt hyperplasia.^205–207^ In neonatal mice, treatment with *L. helveticus* and *L. rhamnosus* reduced colonic crypt hyperplasia and mortality,^208,209^ induced anti-inflammatory pathways, and reduced *C. rodentium* attachment to colonic cells.^209^ Other *Lactobacillus* strains were also shown to protect against A/E pathogens *in vitro* by upregulating secretion of the mucin MUC3 and thereby reducing EPEC adherence.^210^ Preventive treatment with *Bifidobacterium breve* in mice also reduced crypt hyperplasia even if it did not reduce colonization or A/E lesion formation.^211^ However, another report showed a reduction in *C. rodentium* loads in the gut when treating mice with *B. breve* through a mechanism involving production of exopolysaccharide.^212^ Germ-free mice colonized with neonatal microbiota are unable to clear *C. rodentium* infection, but administration of *Clostridia* commensals allowed establishment of CR and clearance of the infection.^197^ Moreover, segmented filamentous bacteria (SFB) have been shown to confer protection against *C. rodentium* infection via promoting protective immune responses in the gut^213^ and stimulating retinoic acid responses in intestinal epithelial cells.^214^

Although many mechanisms conferring CR through the microbiota involve modulation of the immune response,^174,191,206–209,212,213^ microbiota can also have direct effects on *C. rodentium. L. rhamnosus, L. acidophilus*, and *Citrobacter amalonaticus* showed inhibitory effects on *C. rodentium* growth *in vitro*.^206,217^ *C. amalonaticus* also impaired the growth of *C. rodentium in vivo*.^215^ Butyrate, a metabolite produced in the gut by some commensals, inhibited *C. rodentium* growth *in vitro* and reduced colonization *in vivo*.^193^ Treatment of infected mice with *Saccharomyces boulardii* 2 d after *C. rodentium* inoculation, resulted in inhibition of expression and secretion of EspB and inhibition of secretion and translocation of Tir, thereby reducing the number of mucosal adherent *C. rodentium* and ensuing pathology.^216^

Interestingly, although microbiota can play a key role in CR against *C. rodentium* and in clearance of infection, it is also needed for *C. rodentium* virulence.^198^ Following kanamycin-induced dysbiosis in mice infected with Kan^R^ *C. rodentium*, the pathogen was displaced to the cecal lumen and was able to persist avirulently.^198,215^ This was not observed after vancomycin or metronidazole treatment, suggesting *C. rodentium* relies on specific commensals for colonization.^198^ *Bacteroides thetaiotaomicron*, a major constituent of the microbiota, was also shown to enhance the expression of *C. rodentium* virulence genes during infection.^217^ *B. thetaiotaomicron* also contributed to the loss of the mucosal layer during *C. rodentium* infection,^217^ which leads to increased colonization and mortality in mice.^218^

The use of *C. rodentium*, a natural mouse pathogen, as a model for A/E pathogens has given important insights into the complex interactions that occur between the pathogen, the microbiota, and the host in the context of infection.^162,171,174,182,186^

Given the essential role that the microbiota plays in gastrointestinal infection and disease, moving forward it will be key to consider the factors that affect the composition of the microbial community in the gut in order to study the colonization andvirulence mechanisms of A/E pathogens and ensure reproducibility between facilities.

### Clostridioides difficile

The endospore-forming bacterium *C. difficile* (formerly *Clostridium difficile*^219^) is a leading cause of nosocomial antibiotic therapy-associated diarrhea. In the 2000s, its incidence was rising in Europe and North America, concomitant with increases in disease severity.^220,221^ More recent data revealed currently decreasing numbers in the USA,^221^ while the situation in Europe remains less clear because of national differences in detecting and reporting *C. difficile* infection (CDI) cases.^222^ Still, *C. difficile* remains a formidable pathogen burdening the health-care services of Europe and the USA with annual costs of $3 ^223^ and 4.8 billion,^224^ respectively. Annually, the ECDC reports over a hundred thousand cases in Europe,^225^ while the CDC reports just under half a million cases in North America, both with mortality rates of 3–17%.^224,226^ Infection occurs via the fecal-to-oral route through endospores and is facilitated by low hygiene standards. The disease is not only transmitted in health-care facilities but also commonly acquired in community settings and may arise from strains present in the patient’s own microbiome.^227,228^ The role of environmental reservoirs and contaminated food as sources of infection remains elusive. In addition to general risk factors for nosocomial infections, including extended hospitalization, advanced age, and comorbidities, CDI is strongly associated with antibiotic therapy that lowers microbiota-mediated CR.^229^ Symptoms of human CDI ranges from asymptomatic carrier status (approximately 4–15% of healthy individuals were estimated to be asymptomatic carriers^230^), fever, abdominal pain, and watery or bloody diarrhea to life-threatening pseudomembranous colitis.^231^ Asymptotic carriers colonized with toxigenic *C. difficile* are 6 times more likely to experience an episode of CDI than non-colonized individuals.^232^ *C. difficile* pathogenicity depends on its main virulence factors, toxins A and B.^233^ A third toxin, the so-called binary toxin, can exacerbate disease symptoms, but its precise function is still unclear.^234^ Antibiotic therapy with vancomycin or preferably fidaxomicin is the first-line treatment against primary CDI, which most commonly resolves the infection, but may also perpetuate intestinal dysbiosis, leading to recurrent CDI in about ~16% to 25% of cases.^235–239^

Healthy gut microbiota of mice usually provides highly protective CR against infection with *C. difficile* (Figure 2). Therefore, in the past, pathogens have been studied in inherently more susceptible host species, such as hamsters, guinea pigs, and rabbits, as well as germ-free rats and mice.^240–243^ For nearly 3 decades, the main CDI model has been the Syrian hamster, although the severity and lethality of the induced disease are drastically increased compared to the situation in humans. More recently, a more representative conventional mouse model has been established in which mice are pretreated with a cocktail of multiple antibiotics to alleviate CR.^244^ The subsequent infection with *C. difficile* induces strong colitis and high lethality in a dose-dependent manner. Strain-level genetic variabilities in *C. difficile* are responsible for variability in symptom severity in mice. This was shown in a study in which manifestations of diarrhea and weight loss depended on the *C. difficile* strain (R20291, VPI 10463 (ATCC 43255), or 630 (ATCC BAA-1382)) and the type of antibiotic pretreatment.^245^ The SPF mouse model has been used more frequently since and has been further refined by reducing the number of administered antibiotics to break CR. Thus, robust infection models could be established by pretreating mice with either cefoperazone in the drinking water,^246^ streptomycin by gavage^247^ (the same pretreatment as in the streptomycin-pretreated *S*. Tm model), or clindamycin by intraperitoneal injection.^248^ Standard infection models are usually inoculated with *C. difficile* spores, the relevant main transmissive form, but mice may also be infected directly with high doses (10^7^ CFU) of vegetative cells, such as sporulation-deficient *spo0A* mutants to study the role of spores.^249–251^ The high variability of antibiotic pretreatment procedures as well as the genetic differences between *C. difficile* strains counter-intuitively do not affect the number of vegetative cells found in the cecum and the amount of toxins that are produced 18 h p.i.^245,252^ However, each antibiotic shapes the community differently after administration and therefore creates a distinct ecological niche. Hence, differential gene expression, especially in catabolic pathways used for simple carbohydrate-molecule acquisition/uptake, was observed in *C. difficile*.^253^ Vegetative cells can be detected after 6 h p.i. in the distal intestine at low concentrations (10^2^–10^3^ CFU/g). After 18 h, colonization expands to the proximal intestine including the stomach and the maximum concentration of 10^8^ CFU/g is reached in the cecum. Despite colonization of the entire gastrointestinal tract, pathology is limited to the cecum and colon.^254^

Germ-free mice can be infected with low-to-moderate doses of spores (10^3^–10^6^ CFU) without inducing lethality,^242^ although this strongly depends on the virulence of the particular *C. difficile* strain and on the susceptibility of the mouse model.^255–257^ In germ-free mice, the *C. difficile* sporulation (and hence transmission) and toxin production are generally higher than in SPF mouse models.^252^ Therefore, germ-free mice can be useful to study the effect of protective microbial species or other factors against CDI.^258^ Mono-association with these species revealed niche competition with *C. difficile*, especially for nutrients, which is also supported by *in vitro* studies.^257,259,260^ Increased sialic acid levels observed in gnotobiotic mice mono-colonized with *Bacteroides thetaiotaomicron* led to increased fecal *C. difficile* densities, demonstrating that not all species of the gut microbiota may be inherently protective.^261^ Further, the addition of the bile acid 7α-dehydroxylating (deoxycholic and lithocholic acid producing) bacterium *Clostridium scindens* to gnotobiotic OligoMM^12^ mice^60^ showed protective effects against *C. difficile* in early-phase colonization.^247^ The strong correlative data obtained in the antibiotic treated SPF mouse model further support the hypothesis that the main contribution of *C. scindens* to CR is causally related to the conversion of primary bile acids, which enhance *C. difficile* spore germination, into 7α-dehydroxylated secondary bile acids, which inhibit vegetative cells of *C. difficile*.^262^ Secondary bile acids were shown to have not only direct negative impacts on vegetative cells but also synergize with *C. scindens*-secreted antimicrobials that inhibit *C. difficile*.^263^ However, the importance of secondary bile acids was challenged in a recent study using Cyp8b1^−/−^ (cholic acid deficient) mutant mice colonized with *C. scindens* that suggested a bile acid-independent antagonistic effect of *C. scindens* toward *C. difficile*.^256^ Competition for Stickland metabolites may explain most of the protective effect of *C. scindens* in this mouse strain, which previously was mainly, but not exclusively,^264^ attributed to its production of 7α-dehydroxylated bile acids.^256^ This finding remains to be corroborated by *in vivo* studies of *bai* gene *C. scindens* mutants (deficient for bile acid 7α-dehydroxylation), which so far have been unavailable.

Fecal microbiota transplantation (FMT) is already commonly used in patients suffering from multiple episodes of recurrent CDI (rCDI) to restore the host–microbiota-mediated colonization resistance with high success rates. In a rCDI mouse model, it was demonstrated that FMT successfully reduces *C. difficile* to undetectable levels.^265^ The restoration of bile acid homeostasis, especially the conversion of primary bile acids to secondary bile acids, seems an important function to restore microbiota mediated CR.^265–267^ Elevated levels of short-chain fatty acids (SCFA) also correlate with enhanced CR against *C. difficile*^268^ and it was shown that FMT fosters SCFA-producers^269^ implying that reintroduction of SCFA-production is an important feature of colonization resistance restoration.^270^

Despite its remarkable efficacy and the recent FDA approval of a first FMT preparation as therapeutic against *C. difficile* infection,^271^ FMT lacks standardization and carries the risk of introducing undetected opportunistic pathogens as well as other unwanted microbiota-related effects^272^ including possible long-term effects on immunity or development of adiposity. However, there is little solid evidence for negative long-term effects to date.^273–276^ On the contrary, most studies report beneficial outcomes. In order to minimize FMT-related risks, efforts to develop defined microbial therapy have been made both in humans^277^ and mice^278^ where *C. difficile* was successfully inhibited with consortia of 6 to 33 species. A rationally designed bacterial consortium of five mucosal sugar utilizers was also able to limit *C. difficile* expansion in the gut of C57BL/6 mice.^279^

### Listeria monocytogenes

*L. monocytogenes* is a Gram-positive foodborne pathogen that is widely distributed in nature and is readily isolated from soil, water, silage, and vegetation.^280^ This wide ecological distribution reflects the ability to adapt to a range of environmental stress conditions, such as low pH, variations in temperature, and elevated osmolarity that are encountered in the natural environment and in the host.^281^ *L. monocytogenes* is the causative agent of listeriosis, an invasive and potentially fatal infection in susceptible animals and humans. Listeriosis is still considered relatively rare; however, its mortality rate (20–30%) is high relative to other food-borne pathogens,^282^ and it is considered a significant public health concern.^283^ The virulence potential of the bacterial strain and the immune status of the host determines the severity of *L. monocytogenes* infection. The most at-risk groups are those with compromised immune systems including pregnant women (leading to spontaneous miscarriage), infants, older adults, and immunocompromised individuals (leading to meningitis or meningoencephalitis).^284^ Combined epidemiological, clinical, and genomic analysis has identified hypervirulent and hypovirulent clonal clusters of *L. monocytogenes*, with CC1, CC4, and CC6 being demonstrably hypervirulent in mouse models of the disease.^285^

*L. monocytogenes* enters the host after ingestion of contaminated food. Pathogenesis involves systemic disease and the crossing of various epithelial barriers, initially the gastrointestinal barrier and subsequently the blood–brain barrier (to cause meningitis) or the feto-placental barrier in pregnant individuals. Entry into gut epithelial cells occurs through the expression of bacterial internal A (InlA) that engages E-cadherin, an eukaryotic cell membrane receptor that ultimately triggers bacterial internalization (Figure 2).^286^ In particular, goblet cells which express exposed E-cadherin represent a targeted site for initial infection and can directly facilitate gastrointestinal transcytosis.^287^ As established primarily using *in vitro* studies, *L. monocytogenes* is internalized into the vacuole in epithelial cells and expression of the microbial pore-forming toxin Listeriolysin O (LLO) and phospholipase A and PlcB rupture the vacuolar membrane to release bacteria into the host cell cytoplasm where they divide rapidly and can move from cell to cell using a mechanism that involves host actin-based motility.^286^ Upon entry into the host gastrointestinal tract, *L. monocytogenes* senses the local environment to regulate virulence gene expression, thereby switching between saprophytic and infectious gene expression patterns. The major regulator of virulence gene expression is positive regulatory factor A (PrfA) which is regulated at the transcriptional and post-transcriptional levels by different environmental signals which include temperature, carbon-sources transported via phosphoenolpyruvate (PEP), carbohydrate phosphotransferase system (PTS) and stress response regulatory proteins such as Sigma.^288–291^ In particular, non-PTS carbohydrates, such as glycerol, enhance virulence gene expression.^292^ Evidence suggests that nutrient metabolism and virulence are tightly co-regulated to control niche-specific virulence in response to environmental nutritional content.^293^

Germ-free mice are more sensitive to *L. monocytogenes* infection than SPF mice, indicating a role for the microbiota in resistance to infection.^294–296^ Mono-colonization of germ-free mice with *Lactobacillus casei, paracasei*, or *saki* strains resulted in increased survival of mice in the probiotic-treated group after 6 d p.i., suggesting enhanced CR.^295,296^ Archambaud et al.^296^ determined that monocolonization by *Lactobacillus casei* and *paracasei* strains in transgenic ECad^hum^ mice (which express the human E-Cadherin as receptor for *Listeria monocytogenes* internalization; see below) provided an environmental signal which promoted adaptation of the pathogen to the gut. However, colonization with *Lactobacillus* strains also enhanced the anti-Listerial interferon response and reduced dissemination of the pathogen in the host. Other mechanisms by which the gut microbiota may protect against *Listeria* infection include local production of bacteriocins^297^ or the triggering of host expression of anti-Listerial defensins such as RegIIIγ.^298^ Recent studies demonstrated that a high fat diet reduced RegIIIγ expression and increased goblet cell number in normal mice infected with murinized *L. monocytogenes* (which binds to the murine version of the cadherin receptor; see below) and concomitantly increased severity of infection.^299^ Interestingly, whilst RegIIIγ is seen to express high activity against *L. monocytogenes*, RegIIIβ is active against *Salmonella* strains but not *L. monocytogenes* indicating potential nuances in responses to different pathogens.^300^

Evidence suggests that *L. monocytogenes* strains may produce bacteriocins locally in the gut to alter microbiota community structure and favor disease progression. Lmo2776 is a bacteriocin produced by *L. monocytogenes* that targets commensal *Prevotella copri* to enhance infection by the pathogen.^301^ Listeriolysin S (LLS) is a protein with characteristics of both hemolysins and bacteriocins which is produced by epidemic strains of *L. monocytogenes*.^302^ It showed bactericidal activity against *Alloprevotella, Allobaculum*, and *Streptococcus*.^303^ LLS is not cytotoxic and acts locally in the gut as a bacteriocin to modify the microbiota and favor gastrointestinal colonization by the pathogen.^304^

Recent work is beginning to determine the key taxa within the microbiota that provide CR against *L. monocytogenes*. By inference from the studies above *Prevotella copri* may be a key contributor to CR as specific targeting of this species enhances colonization by the pathogen.^301^ Feeding mice, a high-fat diet alters the microbiota and enhances *Listeria* colonization, however further work is necessary to determine whether species that are reduced by high-fat diet and known to influence barrier functions (such as *Akkermansia muciniphila*) are important for protection against the pathogen.^299^ Studies have used streptomycin pretreatment in conventionally raised mice to dramatically reduce CR with subsequent analyses of taxa that contribute to reestablishing resistance to *L. monocytogenes*.^305^ Rational selection and subsequent administration of *Clostridium saccharogumia, Clostridium ramosum, Clostridium hathewayi*, and *Blautia producta* prevented a systemic infection with *L. monocytogenes* in germ-free mice.^305^

Information derived from studies investigating the role of microbiota in CR may give rise to novel methods of treating or protecting against infection. A number of novel approaches have included the identification of potential next-generation probiotic strains,^305^ development of engineered probiotic strains that produce bacteriocins^306^ and the development of engineered probiotics that express InlA, InlB, or other *Listeria* adherence proteins (LAPs) to directly compete with the pathogen.^307,308^ Drolia et al. ^308^ showed that the expression of LAP on the surface of a *Lactobacillus casei* strain reduced intestinal colonization of *L. monocytogenes* and protected mice from lethal infection.

There are several animal models that can be used to investigate the role of microbiota in *L. monocytogenes* infection. Normal inbred laboratory mice are relatively resistant to oral infection with *L. monocytogenes* ^309,310^ due to the significantly reduced affinity of InlA for the murine E-cad receptor. However, the efficiency of infection can be increased by transgenic expression of human E-cad in enterocytes of the small intestine in mice^311,312^ or mutagenesis of InlA in *L. monocytogenes* to create engineered (murinized) strains.^313,314^ Nevertheless, a high dose of administered *L. monocytogenes* is still necessary to study the *in vivo* behavior of *L. monocytogenes*.^309,311^ In addition, murinization of the *inlA* gene in *Listeria* promotes targeting of another receptor, M-cadherin, in mice which alters the local inflammatory response relative to the humanized mouse model system.^315^ Other small animal models such as guinea pigs express E-cadherin that resembles human E-cadherin and may represent an effective animal model for oral infection, albeit they are limited by cost.^316,317^ Some studies utilize high-dose *Listeria* infection in regular laboratory mouse strains.^305^ It should be noted that common mouse strains can differ in ability to mount inflammatory responses or to fix complement and in particular A/J and BALB/c/By/J are more susceptible to intravenous and intraperitoneal *Listeria* infection than C57BL/6 or C57BL/10 mice.^318^ Finally, future studies should examine the role of the microbiota in aged or pregnant models in order to more closely mimic those groups of individuals that are more likely to develop human disease. Pregnant mouse or guinea pig models^319^ or aged mouse colonies^320^ have been examined as models for basic *L. monocytogenes* infection.

## Concluding remarks

Our review highlights the importance of an intact and diverse microbiome for protection against enteric disease. Germ-free and antibiotic pretreated mice are susceptible to colonization by all the pathogens mentioned above. But even more subtle approaches, like switching normal mouse diet to western-style diet, allows colonization of conventional mice with at least two (very distinct) pathogens: the Gram-positive *Listeria monocytogenes* and the Gram-negative *S*. Tm. However, to trigger disease symptoms in mice that are similar to those found in humans, it is often necessary to change certain properties of the mouse immune system and/or the surface receptors of the intestinal epithelial cells. (A comprehensive comparison of the mentioned pathogens can be found in Table 1.) We expect that the diversity offered by this set of murine infection models (Table 2) will provide unique opportunities to demonstrate generalizable principles underlying CR and to discover species-specific adaptations. This tool-box is still expanding as indicated by the recent discovery of *Shigella flexneri* infection models in inflammasome-deficient mice.^321^ First attempts to apply the same principles for therapy of one pathogen to another by restoring CR have already been made.^322^ We hope that this review will further accelerate progress toward a better understanding of gastrointestinal infections in order to harness the protective mechanisms to develop preventive measures and new therapies.

**Table 1.** Key features of the pathogens relevant for enteric infection.

Pathogen	Key virulence factors of pathogen	CR (complex microbiota)	Protection by mucus layer	Innate immune responses	Adaptive immune responses	References
*Salmonella* Typhimurium	flagella, TTSS-1, TTSS-2, sii adhesin	Strong	≈10-fold	NLRC4 inflammasome, TLR, MyD88, TNF, IFN	sIgA against O-antigen	Hapfelmeier (2005); Fattinger (2021); Hausmann et al. (2020); Hausmann (2021); Sellin (2014); Barthel (2003)
*Listeria monocytogens*	InlA, InlB, LAP, lmo1413, LLO, PlcA, PlcB, ActA,	Medium to strong	Evidence for mucous-binding	MyD88, NOD2, NLRC4/AIM2/NLRP3 inflammasome, NF-κB, RegIIIγ	CD8 & CD4 (TH1) T cell response	Brandl, K. (2007); Drolia & Bhunia, (2019); Pizarro-Cerda & Cossart (2018), PMID: 30,523,778
*Campylobacter jejuni*	flagella, LOS, HtrA, Cj0268, adhesins, invasins	Strong (only in mice)	No protection provided (mucus can be a chemoattractant)	NLRP3 inflammasome, TLR, MyD88, TRIF, NF-κB, mTOR	Th1 and Th17 lymphocytes responses, IgA and IgG which can be cross reactive to human gangliosides in neuerons	Heimesaat, M. M. et al. (2014); Kim, S. et al. (2018); Schmidt, A. M. et al. (2019); Mansfield, L. S. et al. (2007); Tetmeyer, N. et al. (2021); Sun, X. et al. (2012)
*Escherichia coli/ C. rodentium*	LEE encoded genes, particularly a type III secretion system (T3SS), translocators, intimin and translocated intimin receptor (Tir); non-LEE encoded effectors + Shiga toxin (Stx) for EHEC	Strong for EPEC and EHEC, no CR in inbred mice for *C. rodentium*	10–100 fold reduction of *C. rodentium* burdens in feces and reduction of mortality compared to mice lacking a mucus layer (*Muc2-/- mice*)	Type 3 immunity, IL-22 and IL-17 secretion, antimicrobial peptide secretion	Type 3 immunity, IgG opsonization	Luperchio and Schauer (2001); Mullineaux-Sanders et al. (2019); Mundy et al. (2006); Vallance et al. (2003); Bergstrom et al. (2010); Silberger et al. (2017)
*Clostridoides difficile*	flagella, TcdA and TcdB, binary toxin CDT, Spo0A	Strong	Unknown	NLRP3 inflammasome, TLR 4/5, MyD88, NOD1, IFNγ, antimicrobials, ROS, RNS	sIgA against TcdA and TcdBHM	Smits, W. K. (2016); Johnson, S. (1992); Abt, M. C., et al,(2016)

**Table 2.** Mouse gut infection models.

Model	Microbiota	Alleviation of CR	Mouse strain	Pathogen density in stool	Onset of enteric disease	Histopathology	Method of infection	Volume and CFU for infection	Pathogen strain	References
***Salmonella*****Typhimurium**										
Streptomycin model	Complex, gnotobiotic	Streptomycin (20 mg p.o.; 24 h before infection)	C57BL/6, Balb/c, 129, any other	10^8^–10^10^ CFU/g	10–12 h p.i.	Epithelium erosion, mucus release by goblet cells, granulocyte infiltration, submucosa edema	Oral gavage	50 µL of 5 × 10^7^ CFU	SL1344	Barthel et al. (2003)
Gnotobiotic microbiota model	OligoMM^12^, LCM	Not needed	C57BL/6 (others not tested)	10^6^ CFU/g at d 1; 10^10^ CFU/g by d 4	2–4 d p.i.	Epithelium erosion, mucus release by goblet cells, granulocyte infiltration, submucosa edema	Oral gavage	50 µL of 5 × 10^7^ CFU	SL1344	Brugiroux et al. (2016); Maier et al. (2013)
Germ-free model	None	Not needed	C57BL/6	10^8^ cfu/g 8 h p.i., 10^9^ at 24 h and 10^10^ at 48 h	8–12 h p.i.	Extreme epithelium erosion, due to slow onset of regeneration; mucus release by goblet cells, granulocyte infiltration, submucosa edema	Oral gavage	50 µL of 5 × 10^7^ CFU	SL1344	Lima-Filho et al. (2004)
Western-diet model	Complex	Switch to WD 24 h before infection	C57BL/6 (others not tested)	10^6^ cfu/g at d 1 and 2, 10^7^ to 10^8^ at d 2 and 10^9^ at d 4	3–4 d pi. (high animal to animal variation)	Epithelium erosion, mucus release by goblet cells, granulocyte infiltration, submucosa edema; high mouse-to-mouse variability	Oral gavage	50 µL of 5 × 10^7^ CFU	SL1344	Wotzka et al. (2019)
***Clostridioides difficile***										
Antibiotic cocktail model	Complex	Kanamycin (0.4 mg/mL), gentamicin (0.035 mg/mL), colistin (850 U/mL), metronidazole (0.215 mg/mL), and vancomycin (0.045 mg/mL) for three d via drinking water. 2 d pause. clindamycin (10 mg/kg) intraperitoneally one d prior to infection	C57BL/6	10^8^–10^9^ CFU/g	24 h p.i.	Epithelium erosion, forming ofpseudomembranes, neutrophil and macrophage exsudation, mucosal injury	Oral gavage	10^3^ CFU	VPI 10463 (ATCC 43255)	Chen et al. (2008)
Cefoperazone model	Complex	Cefoperazone (0.5 mg/ml) for five d via drinking water. 2 d pause prior to challenge. Optionally, clindamycin (10 mg/kg) intraperitoneally one d prior to infection	C57BL/6	10^7^ CFU/g	24 h p.i.	Epithelium erosion, forming of pseudomembranes, neutrophil and macrophage exsudation, mucosal injury	Oral gavage	25 µl with 10^5^ CFUor 6*10^7^ CFU	VPI 10463 (ATCC 43255), R20291 or 630 (ATCC BAA-1382)	Castro-Cordova et al. (2016)
Clindamycin model	Complex	Clindamycin (200 μg) by intraperitoneal injection on d −1	C57BL/6	10^7^ cfu/g at d 1; 10^7^–10^8^ cfu/g by d 2,3	24 h p.i.	Epithelium erosion, forming of pseudomembranes, neutrophil and macrophage exsudation, mucosal injury	Oral gavage	10^3^ CFU	VPI 10463 (ATCC 43255)	Buffie et al. (2012)
Streptomycin model	Complex	Streptomycin 100 μL by gavage, 200 mg/mL by gavage on d −1 or 5.0 mg/ml via drinking water for 5 d with 2 d pause prior to infection	C57BL/6	10^7^–10^8^ CFU/g	18 h – 24 h p.i.	Epithelium erosion, forming of pseudomembranes, neutrophil and macrophage exsudation, mucosal injury	Oral gavage	100 µl 10^3^ CFU	DH1916 or 630 (ATCC BAA-1382)	Studer et al. (2016); Jenior et al. (2017)
Gnotobiotic microbiota model	OligoMM^12^	Not needed	C57BL/6	10^7^ cfu/g at d 1; 10^7^–10^8^ cfu/g by d 3	24 h p.i.	Epithelium erosion, forming of pseudomembranes, neutrophil and macrophage exsudation, mucosal injury	Oral gavage	100 µl with 10^3^ CFU	DH1916	Studer et al. (2016)
Germ-free model	None	Not needed	CD-1, Swiss Webster, C57BL/6	10^9^ CFU/g	18 h − 24 h p.i.	Epithelium erosion, forming of pseudomembranes, neutrophil and macrophage exsudation, mucosal injury	Oral gavage	100 µl with 10^9^ CFU(vegetative cells) or 10^2^ CFU(spores)	HUC2-4, VPI 10463 (ATCC 43255) or 630 (ATCC BAA-1382)	Onderdonk et al. (1980); Reeves et al. (2012)
***Listeria monocytogenes***										
Western-diet model	Complex	Western Diet High fat (45% calories from fat) for 13 d prior to infection	C57Bl/6 J	10^9^ CFU/g at d 1; 10^7^ at d 2: 10^6^ at d 3	24 h.p.i.	Increase of goblet cell numbers by high fat prior to infection; reduced inflammatory response in HF diet post-infection	Oral gavage	200 µl with 5 × 10^9^ CFU	EGDm (murinized)	Las Heras et al. (2019)
Germ-free model andmonocolonized mice with *Lactobacillus sakei 2a*	None	Not needed	germ-free NIH mice	1x10^8^ cfu/g at d 1 in both germ-free and monocolonized mice	ND	Increased survival of monocolonized mice; greater evidence of inflammatory lesions in the ileum, cecum and liver of Listeria infected germ-free mice relative to infected monocolonized mice	Oral gavage	100 µl with 1 × 10^8^ CFU	Scott A (wild-type)	Bambirra et al. (2007)
Streptomycin model	Complex	Streptomycin (20 mg p.o.; 24 h before infection)	C57BL/6, Rag1−/−, Ifng−/− and Il17−/−	1 CFU/g at d 1, 10^4^ at d 6 and 10^2^ d 10	3 d p.i.	Iinfection, edema, inflammatory cell infiltration, and epithelial cell shedding	Oral gavage	1 x 10^8^ CFU (volume unclear)	10403s (wild-type)	Becattini et al. (2017)
Mono-colonized model (Lactobacillus)	A single strain: Lactobacillus	Germ-free mice or mice monocolonized with either Lactobacillus paracasei or Lb. casei	germ-free humanized E-Cad-hum mice	10^6^ at d 1 for germ-free; 10^5^ at d 1monocolonized	ND	Enhancement of host micro-RNA expression and downregulation of IL-2 and IL-10 by Lactobacillus monocolonoization during Listeria infection	Oral gavage	200ul with 5 × 10^9^ CFU	EGDe (wild-type)	Archambaud et al. (2012)
***Campylobacter jejuni***										
Hhumanized FMT model	Human gut microbiota from healthy donors	Not needed	C57BL/6 C57BL/10 IL-10 KO	10^8^ cfu/g	2 d p.i. only in IL-10 KO	Moderate to severe hyperplasia, elevated levels of apoptotic cells, moderate inflammatory cell infiltration into the mucosa, moderate goblet cell loss high mouse-to-mouse variability	Oral gavage	300 µl of 10^9^ CFU	ATCC 43431; 81–176; B2	Mansfield et al. (2007); Bereswill et al. (2011); Heimesaat et al. (2019)
Secondary abiotic model	None	Not needed	C57BL/6 C57BL/10 IL-10 KO	10^8^–10^9^ CFU/g	2 d p.i. only in IL-10 KO	Severe hyperplasia, elevated levels of apoptotic cells, marked inflammatory cell infiltration into the mucosa and submucosa, marked goblet cell loss, multiple crypt abscesses, and crypt loss	Oral gavage	300 µl of 10^9^ CFU	ATCC 43431; 81–176; B2	Mansfield (2007); Bereswill (2011); Heimesaat (2022)
Infant model	Complex; higher*Enterobacteriaceae* loads	Not needed	C57BL/6	10^7^ CFU/g at d 1 p.i. 10^4^–10^5^ CFU/g by d 3 p.i.	6–7 d. p.i.	Mild to moderate inflammatory cell infiltration into the mucosa and submucosa, mild to moderate hyperplasia, mild to moderate goblet cell loss long-term tissue damage is also observed	Oral gavage	300 µl of 10^9^ CFU	ATCC 43431; B2	Haag et al. (2012), Haag et al. (2012)
Defined microbiota model	Nonpathogenic Clostridial species, Lactobacillus, Acinetobacter	Not needed	C3H (others not tested)	10^8^–10^9^ CFU/g	No clinical signs of disease	Mild inflammation in the lamina propria, with preservation of the normal tissue architecture.	Oral gavage	200 µl of 5 × 10^8^ CFU	81–176; NCTC 11168	Chang & Miller (2006)
**EPEC/EHEC**										
Germ-free mice (EHEC)	None	Not needed	Swiss-Webster	10^9^ cfu/g by d 1 and until death	4–7 d p.i.	Renal tubular necrosis, necrosis of colonic epithelial cell, neurologic signs or lesions, luminal fluid accumulation in the cecum	Oral gavage	100 µL with 10^2^ to 10^6^ CFU	EHEC and STEC 86–24, EDL933, DEC8B, DEC10B, TW14359, TW04863, MI02-102, MI04-43, MI06-31, and Sakai	Eaton et al. (2008)
antibiotic pretreated mice (EPEC and EHEC)	complex, gnotobiotic	streptomycin (24 h prior to infection); antibiotic cocktail of gentamicin, vancomycin, metronidazole and colistin (for 3 d prior to infection)	C57BL/6, CD-1	10^7^–10^10^ cfu/g	2–3 d p.i.	Loss of epithelial integrity, moderate edema in the submucosa, infiltration of inflammatory cells into the lamina propria in the ileum and the colon for EPEC	Oral gavage	100 µL with 10^9^ CFU	EPEC E2348/69 (serotype O127:H6) and EPEC E2348/69 DescN CVD425	Ledwaba et al. (2020);Wadolkowski et al. (1990)
antibiotic and mitomycin C pretreated mice (EHEC)	complex, gnotobiotic	streptomycin (continuous treatment) and mitomycin C treatment (3 times every 3 hours after infection)	BALB/c	10^9^ cfu/g by d 1 and until d 7	1–2 d p.i. depending on mitomycin C injections	Apoptotic injury in the cryptic area of the intestinal mucosa, injuries in the bone marrow and mesenteric lymph nodes, toxic tubular necrosis	Oral gavage	100 µL with 2–6 × 10^3^ CFU	STEC 89020087, V354, V406, CDC EDL933, V356, TT-18, V20, V50, TB226A, EDL931 and O-1	Shimizu et al. (2003)
Young mice (EPEC)	Complex, probably immature	Not needed	C57BL/6 N	10^3^–10^8^ CFU/g of colon at 4 d p.i. and for 12 d, age-dependent	Not described	Not described	Oral gavage	1 µL with 0.5–1x10^5^ CFU in young mice and 100 µL with 0.5–1x10^8^ CFU in adult mice	EPEC E2348/69	Dupont et al. (2016)
**Citrobacter rodentium**										
Resistant mouse strain (e.g. C57BL/6)	Complex	Not needed	C57BL/6, BALB/c, 129S1/SvImJ, NIH Swiss	10^8^ CFU/g 1–8 d p.i.; 10^9^ cfu/g 8–12 d p.i. and ddecrease until clearance	Not specified	Cell proliferation and increase in colonic crypt lengths, mucosal inflammatory response	Oral gavage	100 µL with 2.5 × 10^8^ CFU	DBS100	Vallance et al. (2003); Crepin et al. (2016)
Susceptible mouse strain (e.g. C3H/HeJ)	Complex	Not needed	C3H/HeJ	10^9^ CFU counts in the colon at d 4 p.i.	Not specified, death by d 6–10 p.i.	Fulid and mucus accumulation in the colonic lumen, increase in colon weights and crypt lengths, mucosal inflammatory response with immune cell infiltration, submucosal hyperemia and mucosal ulceration, enlargement of the distal colon	Oral gavage	100 µL with 2.5 × 10^8^ CFU or 200 µL with 1–2 × 10^9^ CFU	DBS100; ICC169	Vallance et al. (2003); Mundy et al. (2006)
Germ-free mice	None	Not needed	C57BL/6	10^9^ CFU/g from d 2 p.i. and up to 42 d p.i.	Not specified	A/E lesions	Oral gavage	200 µL with 10^9^–10^10^ CFU	ATCC51459; ICC169; DBS120	Buschor et al. (2017);Mullineaux-Sanders et al. (2017); Kamada et al. (2012); Kamada et al. (2015)
